# Supplementary material for: Towards a Survival-Based Cellular Assay for the Selection of Protease Inhibitors in Escherichia coli
Source: BioTech (Basel). 2025 Mar 7;14(1):16. doi: 10.3390/biotech14010016 (PMC11940102; doi:10.3390/biotech14010016)
Supplement: Supplementary file 1 [file biotech-14-00016-s001.zip › biotech-3439147-supplementary.pdf]

Supplementary Materials: Towards a Survival-Based Cellular Assay for the Selection of Protease Inhibitors in *Escherichia coli*

William Y. Oyadomari, Elizangela A. Carvalho, Gabriel E. Machado, Ana Júlia O. Machado, Gabriel S. Santos, Marcelo Marcondes and Vitor Oliveira

Table S1. Oligonucleotides used for the TEVpro Asp81Asn mutation.

Mutation	Oligonucleotides
TEVpro Asp81Asn	FW -GATGGTCGTAACATGATTATTATCCGTATGCCGAAAG
	Rev -GATAATAATCATGTTACGACCATCGATCAGATGCTGTTGC

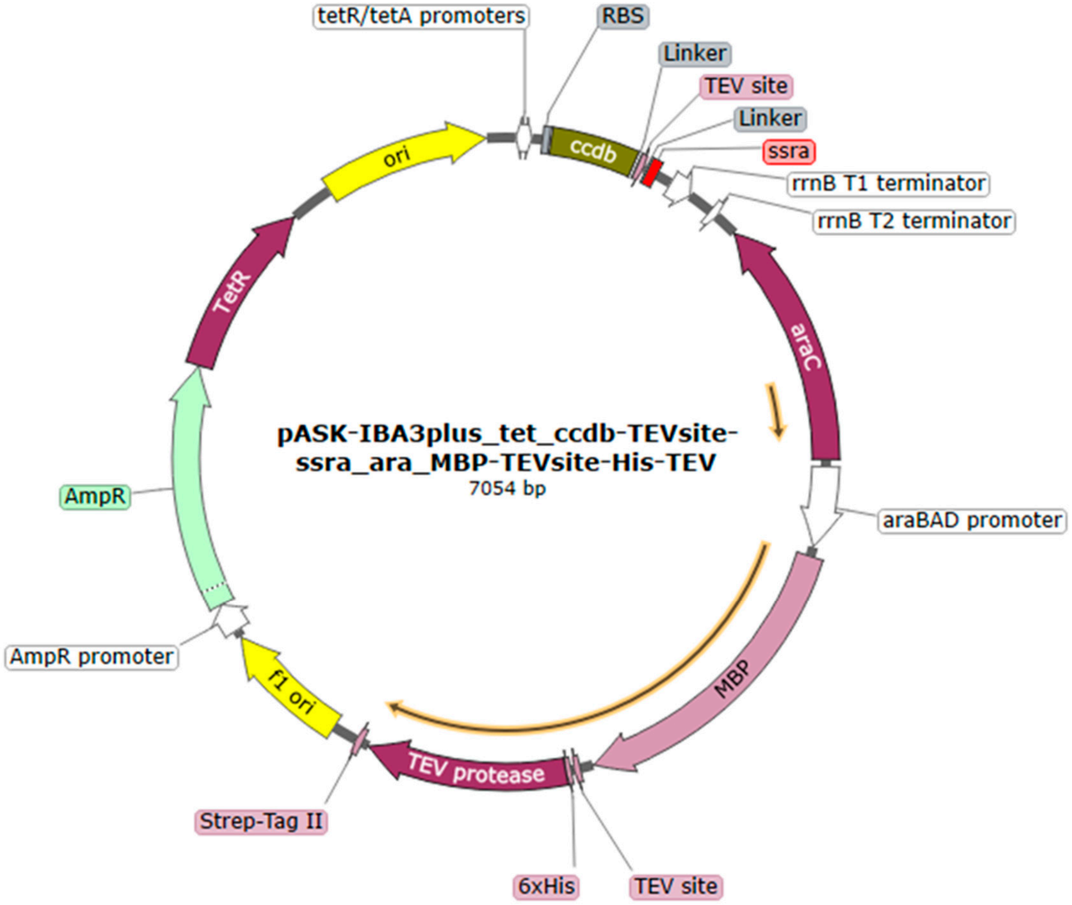

Figure S1. TEVpro vector - plasmid with two independent promoters: The polypeptide chain ccdB-TEV site-ssrA under the control of the Tet promoter and MBP-TEV site-TEV under the control of Ara promoter. Using vector pASk-IBA3plus as a backbone the CCDB – ENLYFQG – ssra substrate gene was cloned under the control of the Tet-sensitive promoter, and the TEVp protease gene in a sequence to be expressed as a fusion protein with the MBP, was cloned under the control of the Ara-sensitive promoter previously introduced to the vector backbone according to the map shown. After initial tests the pBR322 origin was replaced by the ColA origin. The mutation Asp81Asn in the TEV gene made by PCR using the primers shown in Table S1 generated the TEVproi vector (this mutation inactivates the proteolytic activity of the expressed TEV).

A. Amino acid sequence of the **MBP-TEV site-TEV.** The expressed TEV protease contains the mutation S219V that prevents its auto-degradation and improves its stability.

**MKIEEGKLVIWINGDKGYNGLAEVGKKFEKDTGIKVTVEHPDKLEEKFPQVAATGDGPDIIFWAHDRFGGYAQSGLLAEITPDKAFQDKLYPFTWDAV
RYNGKLIAYPIAVEALSLIYNKDLLPNPPKTWEEIPALDKELKAKGKSALMFNLQEPYFTWPLIAADGGYAFKYENGKYDIKDVGVDNAGAKAGLTFLV
DLIKNKHMNADTDYSIAEAAFNKGETAMTINGPWAWSNIDTSKVNYGVTVLPTFKGQPSKPFVGVLSAGINAASPNKELAKEFLENYLLTDEGLEAVN
KDKPLGAVALKSYEEELVKDPRIAATMENAQKGEIMPNIPQMSAFWYAVRTAVINAASGRQTVDEALKDAQTNSSSNNNNNNNNNNLGENLYFQGG
SSRHHHHHHGESLFKGPRDYNPISSTICHLTNESDGHTTSLYGIGFGPFIITNKHLFRRNNGTLLVQSLHGVFKVKNTTTLQQHLIDGRDMIIIRMPKDF
PPFPQKLKFREPQREERICLVTTNFQTKSMSSMVSDTSCTFPSSDGIFWKHWIQTKDGQCGSPLVSTRDGFIVGIHSASNFTNTNNYFTSVPKNFMELLT
NQEAQQWVSGWRLNADSVLWGGHKVFMVKPEEPFQPVKEATQLMNELVYSQ***

B. Amino acid sequence of the **ccdB- TEV site-ssrA** substrate.

**MQFKVYTYKRESRYRLFVDVQSDIIDTPGRRMVIPLASARLLSDKVSRELYPVVHIGDESWRMMTTDMASVPVSVIGEEVADLSHRENDIKNAINLMF
WGIGSGVGENLYFQGSQGGSGAANDENYALAA***

Figure S2. Amino Acid sequencies.

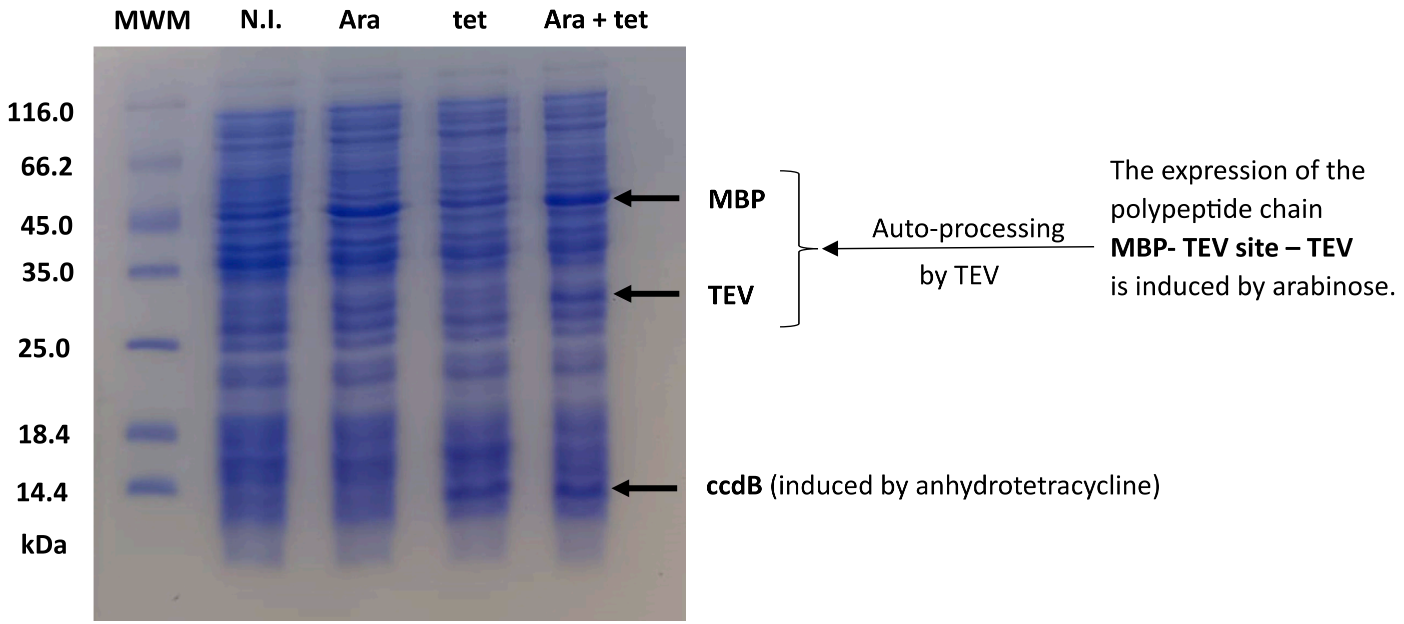

Figure S3. SDS-PAGE analysis. Bacterial extracts from cultures of *E. coli* TOP10 harboring the TEVpro-vector were analyzed.

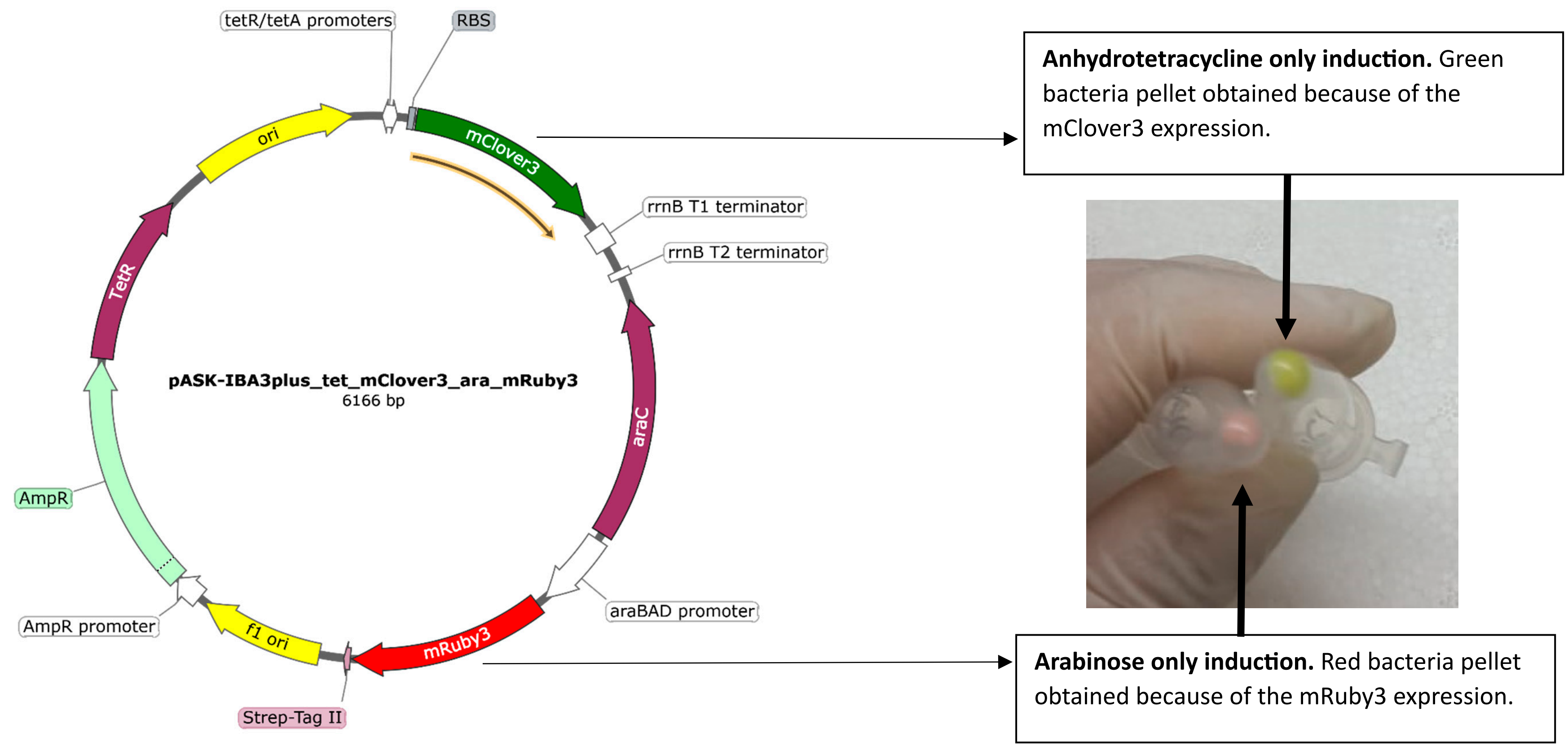

Figure S4. Plasmid vector with two independent promoters expressing mClover3 under the control of the Tet promoter and mRuby3 under the control of Ara promoter. Using vector pASk-IBA3plus as a backbone the mClover3 gene was cloned under the control of the Tet-sensitive promoter, and the mRuby3 gene was cloned under the control of the Ara-sensitive promoter previously introduced to the vector backbone according to the map shown. After initial tests the pBR322 origin was replaced by the ColA origin.

Table S2. Fluorescence intensities of diluted cell extracts from small-scale cultures of E. coli cells harboring the plasmid with mClover3 and mRuby3 genes, which map is shown in Figure 4S, in the presence or in the absence of anhydrotetracycline and arabinose.

	**Fluorescence at λEX = 558 nma λEM = 592 nma**	**Fluorescence at λEX = 506 nmb λEM = 518 nmb**
No induction	25 AUF	278 AUF
After induction	656 AUF	10.184 AUF
Fluorescence Intensity Normalized by the Brightnessc	11	117
	Ratio 1:11 (or ~1:10)	

AUF – arbitrary units of fluorescence. aexcitation and emission wavelength for mRuby3 detection. bexcitation and emission wavelength for mClover3 detection. cbrightness reflects both the molar extinction coefficient and the quantum yield, being the best parameter to compare the intensity of different fluorophores. mRuby3 brightness = 58 and mClover3 brightness = 85 (data from https://www.fpbase.org/).
